# News and Events of the Week

**Published:** 1909-11-13

**Authors:** 


### News and Events of the Week.

At the last meeting of the Board of Dr. Steevens Hospital, Dublin, W. A. Winter, Esq., M.D., F.R.C.P., was appointed Visiting Physician to the hospital, and the following resolution was passed regarding the resignation of H. Jellett, Esq., M.D., F.R.C.P. : "Resolved—That the Governors receive with regret the resignation of Dr. Henry Jellett, and desire to express their sense of his important services to the hospital."

Dr. Stevens, of the Liverpool School of Tropical Medicine, left the Mersey on November 3 by the steamer *City of Venice* on his way to Egypt, where he will undertake a mission on behalf of the school. He will make a special study of helminthology and will investigate various thread worms responsible for many tropical diseases, while he will also collect material for teaching purposes. A special fund is being raised to meet the expenses of this the twenty-third expedition sent out by the Liverpool school.

Dr. E. W. Hope, Medical Officer of Health for Liverpool, delivering the principal address at the annual meeting of the Association of Public Vaccinators, said that in the matter of vaccination important lessons might be learned from Germany. In that country vaccination was compulsory and both primary and secondary vaccination were carried out rigorously and efficiently. Germany's international frontiers were more difficult to defend than our own seaports from an incursion of smallpox but notwithstanding that Germany with a population almost twice as large as that of England had during a recent period of twelve years only 600 deaths from smallpox, whilst in England and Wales there were 6,761 deaths—more than ten times the number—during the same period.

The late Dr. Bell Taylor's love for dumb animals, says the *Ophthalmic Review*, is shown by a provision made in his will, which directs his trustees to keep all his animals in comfort, properly housed and fed, so long as they live, and to set aside sufficient part of his property, which is valued at nearly £120,000, to provide for this. Included among the animal pensioners are four horses, one of which was once ridden by Lord Roberts at a review. The Nottingham oculist for several years visited the sands at Scarborough, and, picking out the horse he considered to be in the worst condition, sent it by train to Nottingham, where he kept it throughout the winter, returning it just prior to the following season to the rightful owner, free of every expense.

In his annual report, Dr. D. L. Thomas, medical officer of health of Stepney, reports the discovery of human bones at 167 Whitechapel Road, where excavations are in progress. The remains were found at a depth varying from two to eight feet, and consisted of bones belonging to at least 80 bodies. No evidences of coffins were discovered, but a farthing dated 1733 was unearthed. From the presence of the farthing, and from other evidence, Dr. Thomas infers that an old plague pit in the immediate neighbourhood had been disturbed at some time after the year 1733, and the contents re-buried at the rear of No. 167. The plague of 1665 reached the East End through Clerkenwell and Shoreditch. In three weeks there were 2,006 deaths in the parish of Stepney, and 1,206 in the parish of Whitechapel. At that time the district was sparsely populated, and on account of the number of bodies that had to be buried, and the difficulty of carting them, the plague pits were placed close to the main road.

The Government of the United States has appointed a commission to investigate the prevalence of pellagra in the Southern States. The disease was first reported in Alabama in 1907, and the authorities now estimate the number of cases at over 5,000.

Surgeon-Lieutenant-Colonel George Richard Turner Phillips, of Folkestone, and late of 28 Palace Court, W., formerly surgeon to the Great Western Railway Company, and to his Majesty's ship *Essex*, left estate valued at £11,333 gross, and £10,925 net.

A curious "death by misadventure" was recorded on Saturday at a Greenwich inquest on Dr. Thomas Guy Alexander, of East Greenwich. The previous Wednesday evening he called his wife into the surgery and asked her to send for a doctor as in mistake for gin he had taken an overdose of strychnine. He died the same evening.

The formal document relating to the admission of women practitioners to membership and fellowship of the R.C.S. has now been signed by all the members of the Council who were present when the by-law was enacted on April 2 of this year, and by the Home Secretary, and it now only remains for the Lord Chancellor and the Lord Chief Justice to append their signatures. The necessary formalities will be completed in time for women to enter for the examinations to be held in January next.

The *Lutheran* publishes the following : A physician of standing, whom we consulted on the ravages of the Christian Scientist fad, took occasion to remark that many of the gentlemen of his profession were acting most unwisely in permitting themselves to be called in at almost the last moment when the Christian Scientist is about to die, in order to sign the death certificate, which is then accepted by the Board of Health, and deceased is recorded as having died with full benefit of physician, if not of clergy. Thus the physicians play into the hands of those who are decrying their own profession, and the new sect is able to escape the odium of a Christian Scientist death. If the physicians were to set their faces against this practice as one man, the health officers would be able to hold post-mortems on the victims of the fad, and thus fetch out into the light the sad tale of deaths and frauds. As it is, the world never hears of the deaths and the delusion goes on.

Under the auspices of the Royal Sanitary Institute, 90 Buckingham Palace Road, S.W., a sessional meeting will be held at Nottingham on November 20, when a discussion on "The Improvement of City Slums by Housing Reform and Otherwise," will be opened by Alderman T. J. Dabell, M.R.C.S., J.P., chairman, Health Committee. In the afternoon it is proposed to visit Radford, Sneinton, and Meadows, districts in which Housing Reform schemes have been carried out or are in progress. The special course on food and meat inspection, arranged for Army officers and professional men, will commence on November 11. The new museum providing greater accommodation, the Committee are arranging for short exhibitions of sanitary apparatus. The first exhibition will be working models of sewage distributors and sprinklers, shown in action, and will be open for inspection from November 10 till the end of January, 1910. The Council has accepted an invitation from the Town Council of Brighton to hold the next Congress and Exhibition of the Institute in Brighton during the first two weeks of September, 1910.

On Tuesday, Wednesday, and Thursday, November 23, 24, and 25, there will be held in the large Caxton Hall an exhibition of Leadless Glazed China and Earthenware. The object of this exhibition is to popularise further the use of ware manufactured without risk to the worker, and thus at once to encourage experiment in leadless glazes and to diminish suffering from lead-poisoning among those employed in potteries. H.R.H. the Princess of Wales is extending to this exhibition the patronage she gave to its predecessor.

The medical profession gains an addition to its limited number of representatives in the House of Commons by the return of Dr. Charles O'Neill for South Armagh. Dr. O'Neill graduated in medicine and surgery in the University of Glasgow, but retired from practice some years ago. As a member of the Coatbridge Town Council for a considerable period he has done excellent service, and is in high repute in Coatbridge itself, where he lives. He has unsuccessfully contested the same division once before, when he was defeated by another Nationalist candidate.

The Council of the Metropolitan Counties Branch of the British Medical Asociation has made arrangements to hold a meeting at which the three direct representatives for England and Wales on the General Medical Council (Drs. H. Langley Browne, H. A. Latimer, and L. G. McManus) will address their constituents who reside in London and its neighbourhood. The meeting will be held on Monday, November 22, at 4.30 p.m., at the St. James's Vestry Hall, Piccadilly, W. (close to Piccadilly Circus). Dr. J. Ford Anderson, the President of the Branch, will preside. All medical men are cordially invited to be present.

The Royal Society of Medicine (Odontological Section) has made the following (among other) regulations for grants in aid of scientific research : (1) The grants exist for the furtherance of scientific research in connection with dentistry. (2) All grants shall expire on May 1 next following the date on which they are made, when a statement of expenditure shall be forwarded to the Hon. Secretary, 20 Hanover Square, W. (3) No part of any grant shall be applied in payment of personal expenses in connection with the research for which the grant has been made, unless a resolution to that effect has been duly passed by the committee. Every recipient of a grant shall be deemed to have notice of these rules. (4) That when the whole of the expense of the inquiry has been provided by the committee the result of the investigation shall be the property of the Odontological Section of the Royal Society of Medicine.

The Directors of Bovril, Limited, received a distinguished party of scientists on Saturday at the Bovril Factory in Old Street, E.C. The company included the members of the South-Eastern Section of the Sanitary Association. The visitors were conducted over the magnificent premises of the company, and commented upon the cleanliness and brightness of the factory and the smart appearance of the factory hands. The splendidly-equipped laboratories, where the raw materials are subjected to the scrutiny of experts, were then inspected, and the visitors were shown the concentrated beef materials in the form in which they arrive direct from the factories of the vast Bovril estates of some ten million acres. In the milling-room was seen the preparation of the albumen and the fibrin—the feeding part of the beef—and it was interesting to note that during the whole process of manufacture, Bovril is never once touched by hand.

A committee of inhabitants of Grimstad, a small town on the south-east coast of Norway, is appealing for subscriptions to purchase the house at Grimstad in which Ibsen lived from 1844 to 1850. Ibsen went to Grimstad as assistant to a chemist named Reimann, and lodged in his house, which is a small wooden building, two stories high. It was during his stay at Grimstad that Ibsen wrote his first play, *Catilina.*

